# Therapeutic Effects of Prostaglandin E2 Receptor Subtype 3 Blockade on Spinal Cord Injury-Induced Detrusor Overactivity

**DOI:** 10.5152/tud.2026.26041

**Published:** 2026-09-10

**Authors:** Lin-Chen Lv, Xiao-Jian Xu, Dong-Yue Guo, Li-Peng Chen, Yao-Feng Zhu, Ben-Kang Shi, Yan Li

**Affiliations:** 1 Department of Urology, Qilu Hospital of Shandong University, Shandong University, Jinan, China; 2 University of Health and Rehabilitation Sciences, Qingdao, Shandong, China; 3 Shenzhen Research Institute of Shandong University, Shenzhen, China

**Keywords:** Prostaglandin E2 receptor subtype 3, detrusor overactivity, spinal cord injury

## Abstract

**Objective:**

Detrusor overactivity (DO) is a severe urological disorder in spinal cord injury (SCI) patients. Conventional pharmacological interventions, such as antimuscarinic agents, show limited therapeutic efficacy and are often associated with adverse effects. Emerging evidence indicates that prostaglandin E2 (PGE2) and its E-prostanoid 3 (EP3) receptor play a pivotal regulatory role in bladder function. This study aimed to investigate whether EP3 receptor antagonism can alleviate DO after SCI.

**Methods:**

Bladder PGE2 and cyclooxygenase-2 levels were assessed after SCI. Urodynamic alterations were evaluated by cystometry. The EP3 receptor antagonist L-826266 (0.1 or 1.0 mg/kg) was intravenously administered to SCI rats. Urodynamic parameters, bladder cyclic adenosine monophosphate levels, histopathological changes, and preliminary systemic safety parameters were further assessed. L-826266 was also tested in PGE2-induced and acetic acid-induced overactive bladder (OAB) models and in healthy rats.

**Results:**

SCI increased bladder PGE2 and cyclooxygenase-2 levels and induced non-voiding contractions (NVCs) during bladder storage phase. Both doses of L-826266 (0.1 and 1.0 mg/kg) increased the intercontraction interval, bladder capacity, and voiding efficiency and reduced NVC frequency in SCI rats. L-826266 also increased bladder cyclic adenosine monophosphate levels without obvious systemic toxicity or histopathological aggravation. In PGE2-induced OAB rats, L-826266 increased bladder capacity and reduced NVC frequency, whereas it had minimal effects on acetic acid-induced OAB. In healthy rats, only 1.0 mg/kg L-826266 prolonged the intercontraction interval and increased bladder capacity.

**Conclusion:**

EP3 receptor antagonist significantly reduced DO in SCI rats, which provides novel insights into the therapeutic agents for bladder dysfunction after SCI.

**Main Points**

- Spinal cord injury increased bladder prostaglandin E2 and cyclooxygenase-2 levels and induced detrusor overactivity.
- E-prostanoid 3 receptor antagonist L-826266 alleviated spinal cord injury-induced detrusor overactivity and increased bladder cyclic adenosine monophosphate levels.
- L-826266 improved prostaglandin E2-induced, but not acetic acid-induced, bladder overactivity.

## Introduction

Spinal cord injury (SCI) results in permanent motor dysfunction and multiple organ dysfunction, causing a heavy economic burden on patients.^1,2^ Urological complications, including detrusor overactivity (DO) and detrusor sphincter dyssynergia (DSD), are prevalent among patients with SCI.^3,4^ Failure to achieve effective control of lower urinary tract dysfunction may result in subsequent upper urinary tract damage, thereby seriously compromising patients’ quality of life. However, conventional pharmacological interventions, such as antimuscarinic agents, show limited therapeutic efficacy and are often associated with adverse effects, such as dry mouth and constipation.^5,6^ Thus, identifying novel therapeutic targets is essential for managing bladder dysfunction in patients with SCI.

Prostaglandin E2 (PGE2) serves as a primary metabolite derived from arachidonic acid through cyclooxygenase-2 (COX-2)-mediated oxidation and is closely involved in the pathogenesis of bladder dysfunction.^7^ Studies have shown that the level of PGE2 in bladder is increased after SCI.^8^ Moreover, PGE2 can induce DO either by directly activating EP receptors or by sensitizing capsaicin-sensitive afferents.^7,9^ Among the EP receptor subtypes, the E-prostanoid 3 (EP3) receptor is abundantly expressed in both the detrusor muscle and bladder afferent fibers.^10^ The EP3 receptor is mainly coupled to Gi/o-dependent signaling and can inhibit adenylate cyclase activity, thereby reducing intracellular cyclic adenosine monophosphate (cAMP) levels.^7^ Accumulating evidence indicates that EP3 receptor inhibition can effectively ameliorate urinary frequency symptoms in overactive bladder (OAB).^11,12^ Nevertheless, the specific role of the EP3 receptor in DO after SCI remains poorly clarified.

In the present study, we first characterized urodynamic changes after SCI and assessed bladder PGE2 and COX-2 levels. We then investigated whether the EP3 receptor antagonist L-826266 could alleviate SCI-induced DO and examined its effects on bladder cAMP signaling, histopathological changes, including inflammation and fibrosis, and preliminary systemic safety parameters. To further assess its therapeutic applicability, we evaluated the effects of L-826266 in PGE2-induced and acetic acid-induced OAB models. Finally, L-826266 was administered to healthy rats to explore the physiological role of EP3 receptors in regulating bladder function. Collectively, our findings offer evidence supporting the therapeutic potential of EP3 receptor antagonist L-826266 for the treatment of neurogenic bladder dysfunction.

## Material and Methods

### Animals

A total of 136 eight-week-old female Sprague–Dawley rats were kept in cages lined with soft bedding. The animal room adopted a 12-hour reversed light–dark cycle, and all rats had free access to food and drinking water. The study complied with the National Institutes of Health (NIH) guidelines for laboratory animal use and Animal Research: Reporting of *In Vivo* Experiments (ARRIVE). This research was approved by the Institutional Animal Care and Use Committee of Shandong University (Approval No. 26017; start date: April 17, 2026). This study was carried out from April 17, 2026, to July 19, 2026. Animals were assigned to each group randomly using a computer-generated randomization sequence after eligibility criteria were confirmed. The number of animals included in each group is described in the corresponding figure legends.

### SCI

Due to its relatively simple surgical procedure and stable phenotypic characteristics, the rat model of complete SCI was adopted in this study, with the modeling protocol consistent with that described in previous studies.^13^ All surgical procedures were performed under sterile conditions to minimize the risk of postoperative infection. Isoflurane (RWD, Shenzhen, China, R510-22) was applied for rat anesthesia; after confirming adequate anesthesia, rats were placed in a prone position and the dorsal skin over the thoracic vertebrae (T9-T10) was shaved and disinfected sequentially. A 2-cm midline incision was made along the dorsal skin at the T9-T10 level, and the subcutaneous connective tissue and muscles were bluntly dissected to expose the T9 and T10 vertebral laminae. After laminectomy, complete SCI was induced by complete transection at the T9-T10 of the spinal cord using fine sterile scissors (Figure 1A). Sterile gauze was gently applied to the injury site to achieve hemostasis. The incision was irrigated with sterile saline, followed by layered suture and iodophor disinfection. Postoperatively, rats were placed on a heating pad to maintain body temperature until recovery. An antibiotic regimen consisting of daily intraperitoneal penicillin was maintained for 3 days, and regular replacement of bedding combined with twice-daily manual bladder expression (Credé’s maneuver) was performed to maintain hygiene and avert lower urinary tract infections. SCI initially induces a bladder areflexia phase with abolished spinal reflex activity and urinary retention. Subsequently, involuntary reflex contractions of the bladder detrusor are triggered by visceral stimuli. Therefore, urodynamic evaluation was conducted on day 21 after SCI, a time point when prominent non-voiding contractions (NVCs) could be observed.

**Figure 1.**
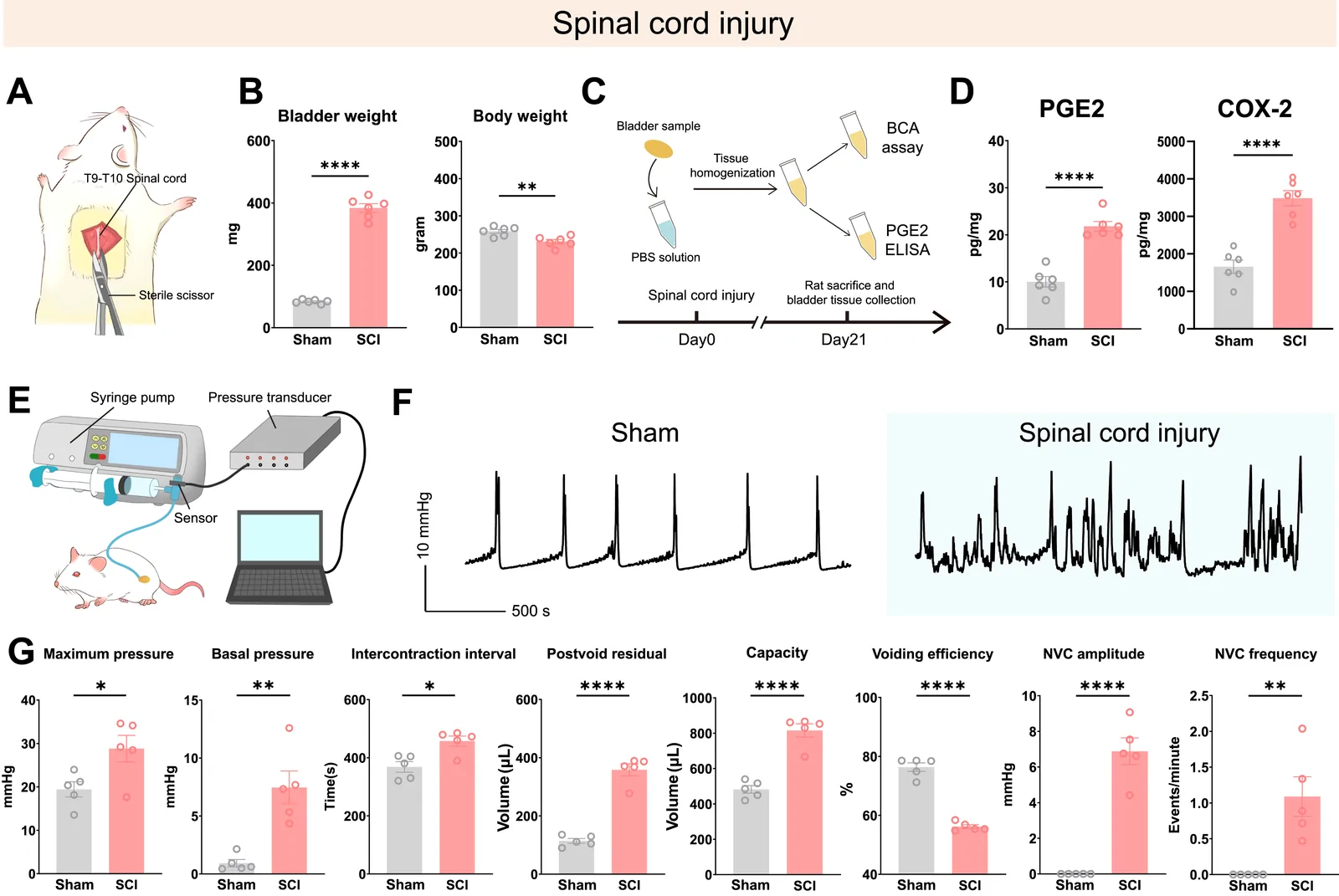
Increased bladder COX-2/PGE2 levels and prominent DO in SCI rats. (A) Schematic diagram of rat SCI modeling. (B) Measurement of bladder weight and body weight in rats at 21 days after SCI (n = 8 per group). (C) Schematic flow chart of the detection procedure for PGE2 content in the bladder. (D) Determination of bladder PGE2 content by ELISA, unit: pg/mg protein (n = 6 per group). (E) Schematic diagram of urodynamic examination. (F) Representative urodynamic curves of rats in the sham group and rats 21 days after SCI. (G) Changes in statistical urodynamic parameters in rats following SCI (n = 5 per group). Data are shown as mean ± SEM. *P < .05, **P < .01; ***P < .001; ****P < .0001; ns, not significant. COX-2, cyclooxygenase-2; DO, detrusor overactivity; ELISA, enzyme-linked immunosorbent assay; PGE2, prostaglandin E2; SCI, spinal cord injury; SEM, standard error of the mean.

### Cystometry

Cystometry was carried out on rats anesthetized with urethane (1.2 g/kg) as previously described.^14^ A PE-50 tube (RWD, Shenzhen, China, No. 62327) was implanted into the bladder dome, through which room-temperature saline was delivered at a rate of 60 µL/min.^15,16^ Data were recorded using a PowerLab system (ADInstruments, Bella vista, Australia). During cystometry, urodynamic parameters were recorded from 3 stable consecutive waveforms. Consistent with prior reports, NVCs were identified as NVC of the detrusor muscle with an amplitude higher than 5 cmH_2_O (3.675 mm Hg).^17,18^ The intercontraction interval (micturition interval) was defined as the interval between 2 consecutive voiding contractions. For cystometric data analysis, the investigator responsible for extracting and quantifying urodynamic parameters was blinded to the animal group allocation and treatment condition. Group identities were decoded only after completion of data analysis.

### OAB Model

To establish the OAB rat model, 0.25% acetic acid (MedChemExpress, Monmouth Junction, NJ, USA, HY-Y0319, dissolved in saline) was continuously instilled into the bladder at 60 µL/min for 45 minutes. Following this induction period, the acetic acid infusion was terminated, and the bladder was maintained with continuous saline infusion.^19,20^ A stock solution was prepared by dissolving PGE2 (MedChemExpress, Monmouth Junction, NJ, USA, HY-101952) in ethanol and stored at −20°C. For subsequent experiments, an aliquot of the stock solution was diluted with saline to a final concentration of 100 µM, which was then continuously infused into the bladder for at least 1 hour.^21,22^

### EP3 Receptor Antagonist L-826266 Administration

L-826266 (MedChemExpress, Monmouth Junction, NJ, USA, HY-19361) was utilized as the EP3 receptor antagonist. According to the manufacturer’s instructions and previous studies,^23,24^ a stock solution was prepared by dissolving L-826266 in DMSO. Before administration, rats were weighed, and then required doses of 0.1 or 1.0 mg/kg were prepared by diluting the stock solution with saline. During cystometry, after stable bladder contractions were established, the rat’s tail was disinfected with 75% ethanol. The L-826266 solution was then administered via tail vein injection. Urodynamic data were recorded both before and after the injection to evaluate whether L-826266 could modulate bladder function.

### Bladder Tissue Collection and Preparation

Following euthanasia, bladder tissues were carefully isolated for biochemical and histological analyses. Briefly, the urethra was transected at the bladder neck. The bladder was collected and gently rinsed with phosphate-buffered saline solution to clear residual urine contents. For enzyme-linked immunosorbent assay (ELISA), bladder tissues were flash-frozen in liquid nitrogen and kept at −80°C until further processing. For histological examination, bladders were immersed in 4% paraformaldehyde overnight, followed by standard dehydration, paraffin embedding, and slicing.

### ELISA

Tissue samples were homogenized with phosphate-buffered saline solution in accordance with the protocols of the PGE2 ELISA kit (Cloud-Clone, Wuhan, China, No. CEA538Ge), COX-2 ELISA kit (Elabscience, Wuhan, China, No. E-EL-R0792), and cAMP ELISA kit (Elabscience, Wuhan, China, No. E-EL-0056). After centrifugation, the collected supernatants were prepared for ELISA. Total protein quantification of ELISA samples was performed using the BCA kit (Servicebio, Wuhan, China, No. G2026), and PGE2 levels were finally normalized to total protein content and calculated as pg/mg (for PGE2 and COX-2) or ng/mg (for cAMP) protein.

### Electrocardiogram

Electrocardiographic monitoring was conducted at 21 days post-SCI following a previously described protocol.^13^ Briefly, rats were anesthetized with isoflurane. Surface electrocardiogram (ECG) signals were obtained using electrodes positioned on the bilateral forelimbs and the right hindlimb. Heart rate was continuously acquired and analyzed using a PowerLab data acquisition system (ADInstruments, Bella vista, Australia).

### Blood Pressure Measurement

Hemodynamic parameters were evaluated at 21 days after SCI according to previously reported procedures.^13^ A PE-50 catheter was inserted into the right carotid artery under isoflurane anesthesia to monitor arterial pressure. Subsequently, a PowerLab data acquisition system (ADInstruments, Bella vista, Australia) was employed to record systolic blood pressure, diastolic blood pressure, and mean arterial pressure.

### Hematological and Serum Biochemical Analysis

To evaluate the systemic safety profile of L-826266 administration, hematological and serum biochemical parameters were analyzed after treatment. Whole blood samples were collected from rats and subjected to complete blood count analysis using an automated hematology analyzer (BC-2800Vet, Mindray, Shenzhen, China). Red blood cell count, white blood cell count, and platelet count were determined.

For serum biochemical analysis, blood samples were allowed to clot and were centrifuged at 3000 rpm for 15 minutes at 2-8°C. Serum samples were collected for biochemical measurements. Serum levels of alanine aminotransferase, aspartate aminotransferase, albumin, blood urea nitrogen, and creatinine were measured using an automated biochemical analyzer (Chemray 800, Rayto Life and Analytical Sciences, Shenzhen, China). All biochemical parameters were analyzed according to the manufacturer’s instructions.

### Hematoxylin and Eosin (H&E) Staining, Masson’s Trichrome Staining, and Histological Evaluation

Paraffin-embedded bladder tissues were sectioned at a thickness of 5 μm and mounted onto glass slides. Following deparaffinization and rehydration, H&E staining or Masson’s trichrome staining was performed using commercial staining kits (Servicebio, Wuhan, China, G1005 and G1006, respectively). All histological analyses were performed by investigators who were blinded to the experimental groups throughout the evaluation process. Images were randomly acquired from representative regions of each section.

Bladder mucosal edema was evaluated based on previously described criteria. Briefly, 4 representative fields were randomly selected from each section, and edema severity was independently assessed. The scoring system ranged from 0 to 3, representing no edema, mild edema, moderate edema, and severe edema, respectively. The edema score was calculated by summing the 4 scores and dividing the total score by 12 and is expressed as a percentage.

Masson’s trichrome staining was used to evaluate collagen deposition and structural remodeling of bladder tissues. Three representative fields were randomly selected from each stained section, and the collagen-positive area and smooth muscle area were quantified using ImageJ software (National Institutes of Health, Bethesda, MD, USA). The collagen deposition level was expressed as the ratio of collagen-positive area to smooth muscle area, and the mean value obtained from the analyzed fields was used for statistical evaluation.

### Statistical Analysis

All statistical analyses were performed using SPSS Statistics for Windows, version 27.0 (IBM Corp., Armonk, NY, USA). Graphical representations and further statistical evaluations were generated with GraphPad Prism 9.0 (GraphPad Software, San Diego, CA, USA). Differences between groups were determined by an unpaired 2-tailed Student’s *t* test. Comparisons among multiple groups were made using 1-way analysis of variance (ANOVA). Comparisons involving treatment and time were performed using a two-way mixed ANOVA, with treatment as the between-subjects factor and time (before vs. after treatment) as the within-subject factor. Multiple testing corrections were conducted via the Benjamini–Hochberg method to control the false discovery rate. Results are presented as mean ± standard error of the mean, and the significance level was defined as *P* < .05. The authors declared that no artificial intelligence tool was used in the preparation of the manuscript.

## Results

### Changes in Bladder PGE2 Level and Urodynamic Manifestations Following SCI

After SCI, bladder weight was significantly increased compared with the sham group rats, while body weight was markedly decreased (Figure 1B). Elevated PGE2 levels in bladder tissue also serve as a crucial factor in the development of DO.^8^ In this study, ELISA revealed that the bladder PGE2 and COX-2 levels were increased in SCI rats compared with the sham group rats (Figure 1C and D). We then investigated the urodynamic change in SCI rats (Figure 1E). Cystometry results showed that SCI rats exhibited a higher maximum pressure, baseline pressure, micturition interval, post-void residual volume, and capacity, but decreased voiding efficiency (Figure 1F and G). NVCs are rarely observed in the sham group rats, but become evident during the storage phase in SCI rats, with significant elevations in NVC amplitude and frequency (Figure 1F and G). These results indicate that elevated baseline and peak pressure, increased post-void residual volume, reduced micturition efficiency, and the emergence of NVCs are prominent urodynamic characteristics following SCI.

### L-826266 Increased Bladder cAMP Levels Without Obvious Systemic Toxicity or Histopathological Aggravation

Safety assessment was performed after L-826266 administration. No significant changes were observed in heart rate; blood pressure parameters, including systolic blood pressure, diastolic blood pressure, and mean arterial pressure; or hematological and biochemical parameters, including red blood cell, white blood cell, and platelet counts, following L-826266 administration (Figure 2A). Serum biochemical parameters related to hepatic and renal function, including alanine aminotransferase, aspartate aminotransferase, albumin, blood urea nitrogen, and creatinine, also showed no significance after treatment (Figure 2A). To examine the potential downstream effects of EP3 receptor blockade, we measured bladder cAMP levels after L-826266 administration. Both 0.1 and 1.0 mg/kg L-826266 significantly increased bladder cAMP levels in SCI rats compared with vehicle treatment (Figure 2B), indicating modulation of cAMP-related downstream signaling. Bladder histopathology was evaluated to determine whether L-826266 caused obvious tissue injury or aggravated SCI-associated bladder remodeling. Histological assessment showed no apparent urothelial edema or fibrotic changes after acute L-826266 treatment (Figure 2C).

**Figure 2.**
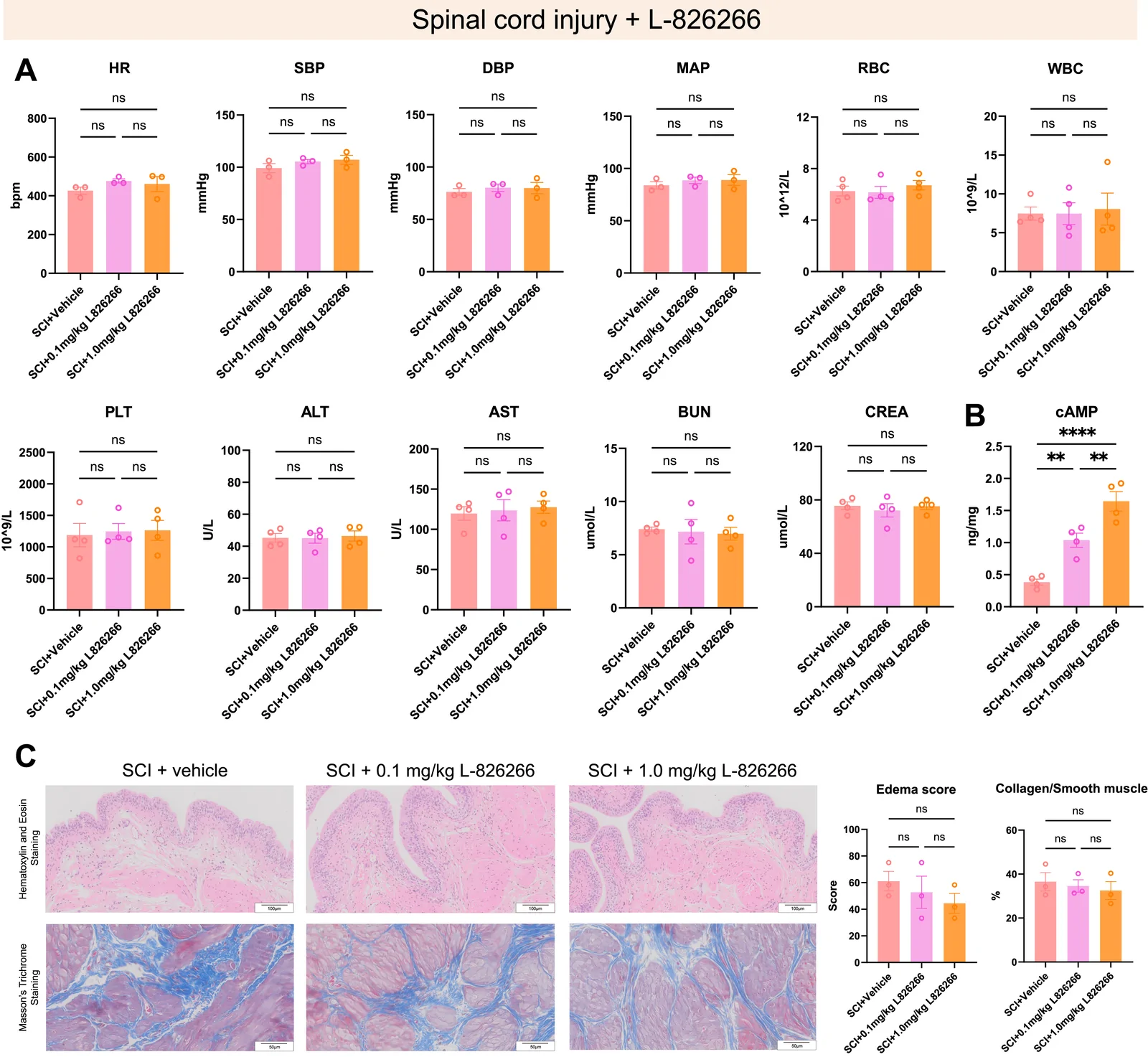
Effects of L-826266 on cardiovascular parameters, hematological and biochemical parameters, bladder cAMP levels, and bladder histological assessment in SCI rats. (A) Effects of different doses of L-826266 on cardiovascular parameters (n = 3 per group) and hematological and biochemical indices (n = 4 per group) in SCI rats. (B) Impact of varying doses of L-826266 on cAMP levels in SCI rats, unit: ng/mg protein (n = 4 per group). (C) Pathological changes in the bladder of SCI rats treated with different doses of the drug (n = 3 per group). Data are shown as mean ± SEM. Statistical differences among groups were analyzed using 1-way ANOVA followed by Tukey’s HSD post hoc test. *P < .05, **P < .01; ***P < .001; ****P < .0001; ns, not significant. Parameters with undetectable values were excluded from statistical comparison. ANOVA, analysis of variance; cAMP, cyclic adenosine monophosphate; HSD, honestly significant difference; SCI, spinal cord injury; SEM, standard error of the mean.

### DO in SCI Rats Was Alleviated Following Treatment with the EP3 Receptor Antagonist L-826266

To investigate whether EP3 receptor antagonist can alleviate DO secondary to SCI, SCI rats were administered the EP3 receptor antagonist L-826266 at a dose of 0.1 or 1.0 mg/kg, and urodynamic tests were performed to evaluate its effect. The results showed that after the administration of L-826266 at both doses, the intercontraction interval, bladder capacity, and micturition efficiency were significantly increased (Figure 3B). Moreover, treatment with the EP3 receptor antagonist decreased the frequency of NVCs during the storage phase (Figure 3B). In addition, no significant differences were observed in other urodynamic parameters before and after L-826266 administration. In conclusion, EP3 receptor antagonist can effectively reduce the NVC frequency and increase the bladder capacity and micturition efficiency in SCI rats, indicating that they can effectively alleviate DO secondary to SCI.

**Figure 3.**
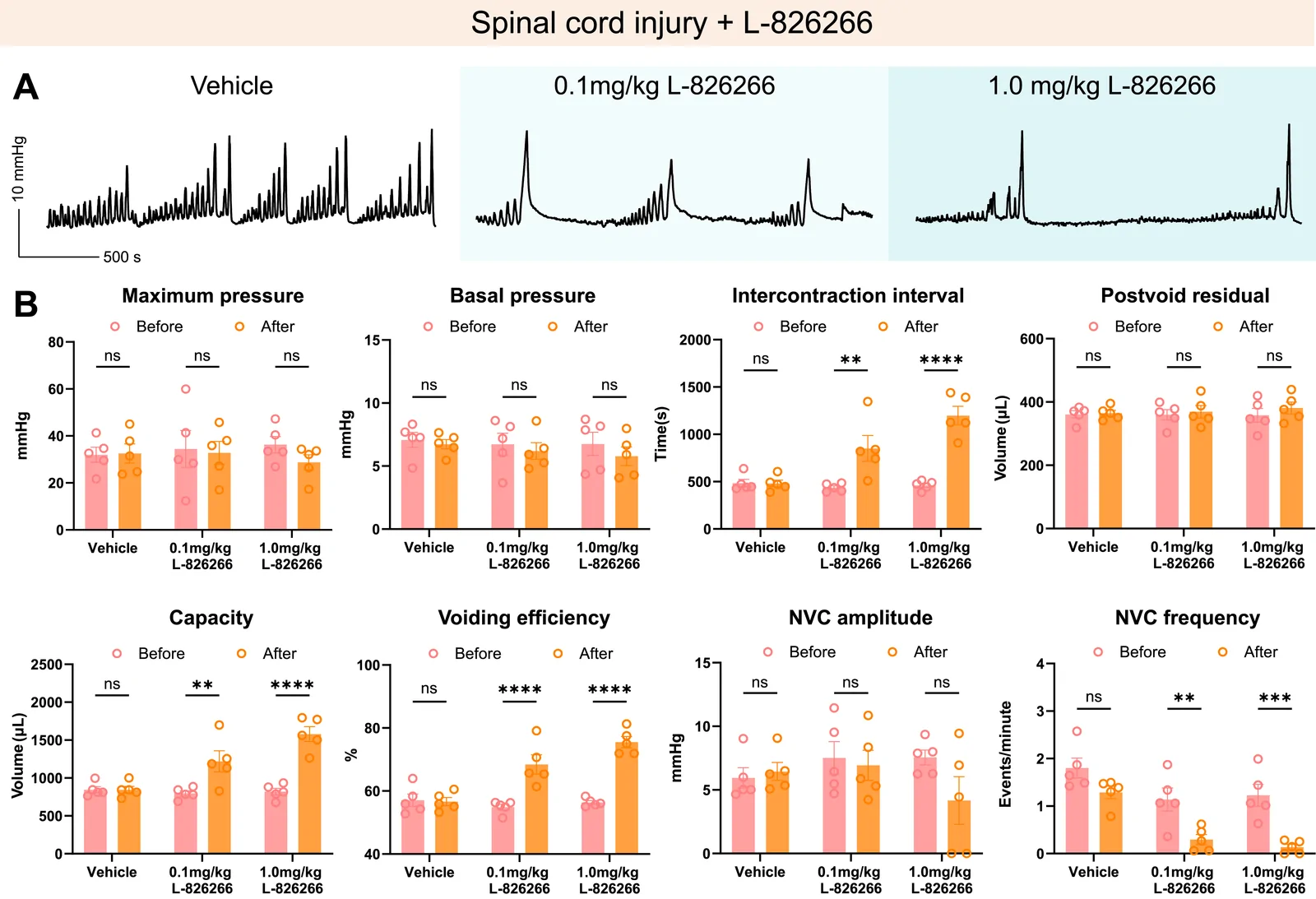
The EP3 antagonist L-826266 alleviated DO in SCI rats. (A) Representative urodynamic curves of spinal cord-injured rats after administration of different doses of the EP3 antagonist L-826266. (B) Changes in urodynamic parameters before and after treatment with different doses of L-826266 (n = 5 per group). Data are shown as mean ± SEM. Two-way ANOVA with the Benjamini–Hochberg FDR correction for multiple comparisons was used for statistical analysis. *P < .05, **P < .01; ***P < .001; ****P < .0001; ns, not significant. ANOVA, analysis of variance; DO, detrusor overactivity; EP3, E-prostanoid 3; FDR, false discovery rate; SCI, spinal cord injury; SEM, standard error of the mean.

### EP3 Receptor Antagonist Alleviates PGE2-Induced Bladder Overactivity But Not Acetic Acid-Induced OAB

In the PGE2-induced OAB rat model, administration of L-826266 at either 0.1 or 1.0 mg/kg significantly prolonged the micturition interval and increased the bladder capacity (Figure 4A and B). Specifically, 1.0 mg/kg L-826266 also improved micturition efficiency and reduced the frequency of NVCs during the storage phase; a similar trend was observed in the 0.1 mg/kg L-826266 group, although the differences did not reach statistical significance (Figure 4A and B). In addition, regardless of the dose of L-826266 administered, no significant changes in other cystometric parameters were observed before and after drug administration (Figure 4A and B). In contrast, in the acetic acid-induced OAB rat model, no significant changes in urodynamic indicators were observed in rats after the administration of L-826266 (Figure 4C and D).

**Figure 4.**
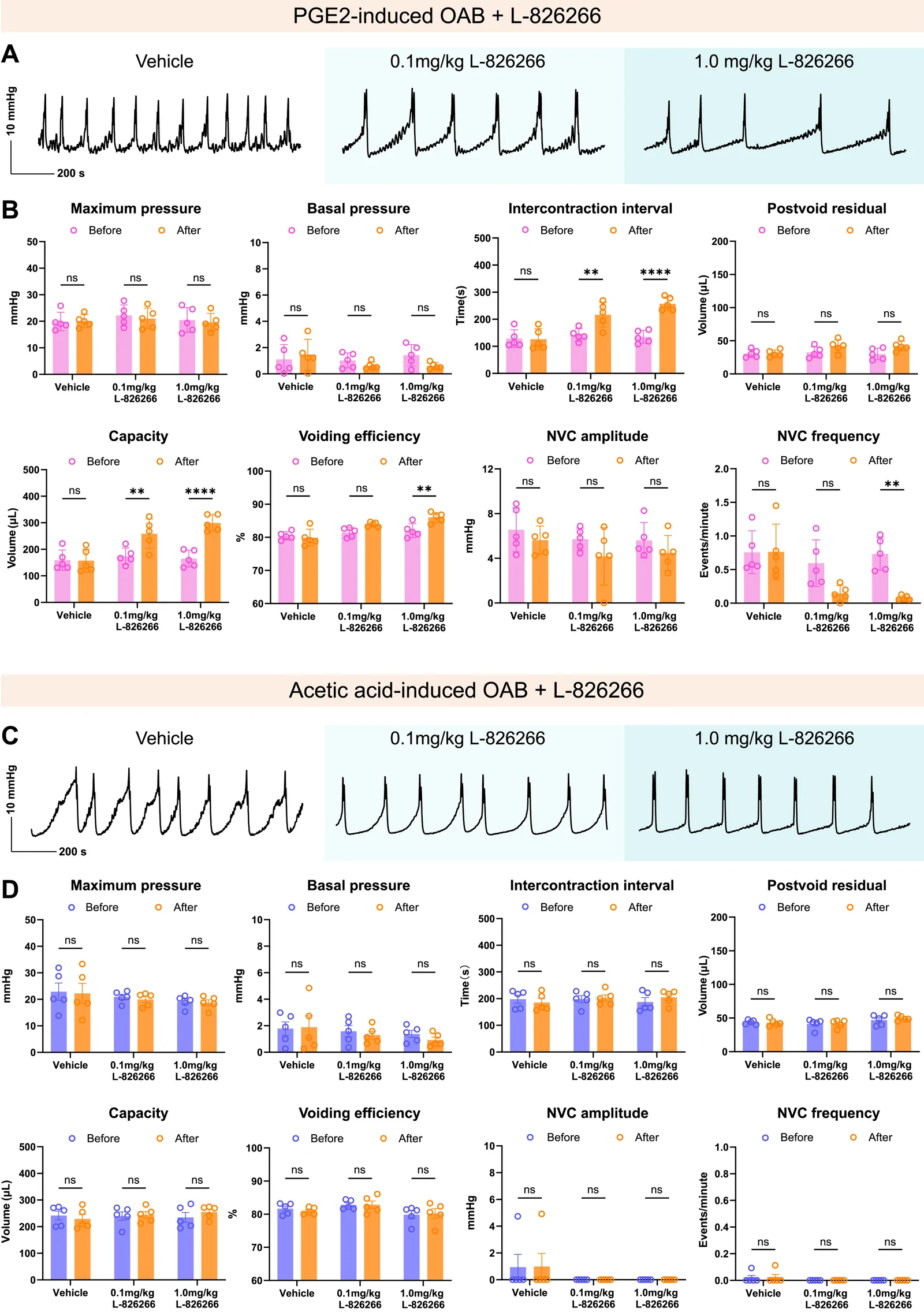
Effects of L-826266 on bladder function in PGE2-induced OAB rats and acetic acid-induced OAB rats. (A) Representative intravesical pressure traces in PGE2-induced OAB rats receiving vehicle, 0.1 mg/kg L-826266, or 1.0 mg/kg L-826266. (B) Effects of different doses of L-826266 on urodynamic parameters in rats with PGE2-induced OAB (n = 5 per group). (C) Representative intravesical pressure traces from rats with acetic acid-evoked OAB following L-826266 treatment at 0.1 and 1.0 mg/kg. (D) Comparisons of urodynamic alterations among acetic acid-challenged OAB rats administered graded doses of L-826266 (n = 5 per group). Data are shown as mean ± SEM. Two-way ANOVA with the Benjamini–Hochberg FDR correction for multiple comparisons was used for statistical analysis. *P < .05, **P < .01; ***P < .001; ****P < .0001; ns, not significant. Parameters with undetectable values were excluded from statistical comparison. ANOVA, analysis of variance; FDR, false discovery rate; OAB, overactive bladder; PGE2, prostaglandin E2; SEM, standard error of the mean.

### EP3 Receptor Antagonist Prolongs the Micturition Interval in Rats Under Physiological Conditions

To explore the physiological role of the EP3 receptors in modulating bladder function, the EP3 receptor antagonist L-826266 was administered intravenously in healthy rats at different doses during cystometry recording. The results showed that under physiological conditions, administration of L-826266 at different doses had no significant effects on the baseline bladder pressure, maximum bladder pressure, post-void residual volume, or micturition efficiency (Figure 5A and B). Specifically, intravenous injection of 1.0 mg/kg L-826266 significantly increased the intercontraction interval and bladder capacity; a similar trend was observed in the 0.1 mg/kg group, although the difference did not reach statistical significance (Figure 5A and B). Furthermore, no NVCs were observed under physiological conditions, regardless of whether L-826266 was administered or not (Figure 5A and B). In conclusion, under physiological conditions, the application of L-826266 at a high dose (1.0 mg/kg) can significantly prolong the micturition interval and increase the bladder capacity, without obvious effects on other basic urodynamic indicators or induction of NVCs.

**Figure 5.**
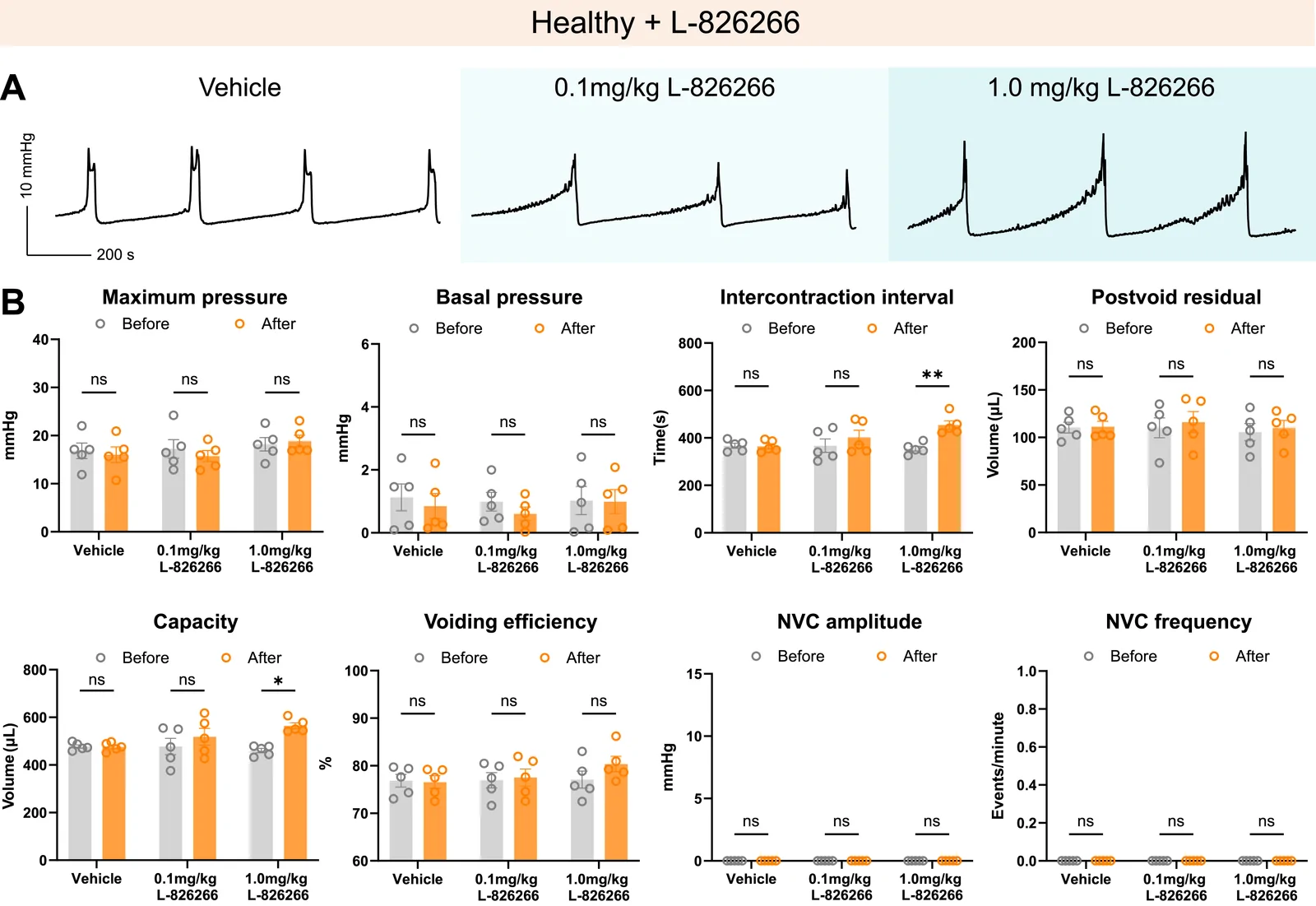
Effects of L-826266 on bladder function in healthy rats. (A) Representative intravesical pressure tracings for the healthy + vehicle group rats, healthy + 0.1 mg/kg L-826266 group rats, and the healthy + 1.0 mg/kg L-826266 group rats. (B) Statistics of urodynamic parameters of maximum pressure, baseline pressure, intercontraction interval, post-void residual, bladder capacity, voiding efficiency, NVC amplitude, and frequency of NVCs across groups (n = 5 per group). Data are shown as mean ± SEM. Two-way ANOVA with the Benjamini–Hochberg FDR correction for multiple comparisons was used for statistical analysis. *P < .05, **P < .01; ns, not significant. Parameters with undetectable values were excluded from statistical comparison. ANOVA, analysis of variance; FDR, false discovery rate; NVCs, non-voiding contractions; SEM, standard error of the mean.

## Discussion

Spinal cord injury frequently results in severe DO. Current guidelines recommend antimuscarinic agents for neurogenic detrusor overactivity.^25^ However, their clinical use may be limited by incomplete efficacy and adverse effects, including dry mouth and constipation.^5,6^ Here, we demonstrated that targeted blockade of the EP3 receptor significantly alleviated DO in rats with SCI.

The pathological process of DO after SCI is generally considered to be mediated, at least in part, by hypersensitive C-fiber afferent nerves, whose abnormal excitation triggers involuntary reflex contractions of the detrusor muscle.^26^ Another important contributor to DO is PGE2 signaling, as Masunaga et al^8^ reported elevated PGE2 production in bladders from SCI rats and demonstrated its role in mediating DO. Consistently, we confirmed increased bladder PGE2 levels after SCI and observed elevated COX-2 expression in bladder tissue from SCI rats. This observation is supported by Park et al,^27^ who reported that mechanical stretch upregulated COX-2 expression in bladder muscle cells and increased PGE2 release into the culture medium. Another study has demonstrated COX-2 upregulation in both urothelial/suburothelial compartments under inflammatory bladder conditions.^28^ In SCI rats, increased post-void residual volume and urinary retention lead to chronic bladder distension and sustained mechanical stretch of the detrusor smooth muscle, which constitutes an important mechanism underlying the elevated PGE2 levels.

PGE2 can induce DO either by directly acting on the bladder smooth muscle or by indirectly modulating neural pathways.^7,10^ Receptors for PGE2, including EP1, EP2, EP3, and EP4, are widely distributed in the urothelium and detrusor muscle, which plays a critical role in regulating bladder function. For example, studies have confirmed that activation of EP3 receptors directly reduces bladder capacity and triggers DO, constituting a key mechanism underlying PGE2-mediated DO.^12,29^ In addition, Maggi et al^9^ reported that the effect of intravesical PGE2 infusion on reducing bladder capacity may depend on the capsaicin-sensitive C-fiber afferent pathway, indicating that sensitizing C-fibers is also indirectly involved in PGE2-induced DO. However, Hokanson et al^30^ demonstrated that intravesical PGE2 still significantly reduced bladder capacity after capsaicin desensitization, suggesting that PGE2-mediated regulation of bladder function is not entirely dependent on capsaicin-sensitive C-fibers. These findings further support that EP receptor-mediated signaling may represent a major pathway through which PGE2 exerts its regulatory effects on bladder activity. We found that the EP3 receptor antagonist increased bladder capacity and decreased NVC frequency in SCI rats without impairing cardiovascular function or hematological parameters, indicating that the PGE2/EP3 signaling axis participates critically in DO progression, which offers a promising therapeutic target for post-SCI DO. Additionally, EP3 receptors have also been studied in lower urinary tract dysfunction resulting from other neurological disorders. In mice with neurogenic bladder induced by encephalomyelitis, the EP3 receptor agonist increased voiding frequency and decreased voided volume, whereas the EP3 receptor antagonist reduced voiding frequency.^31^ These findings suggest that the EP3 receptor may serve as a potential therapeutic target for neurogenic lower urinary tract dysfunction.

We further investigated the therapeutic potential of L-826266 in other models of bladder dysfunction and its regulatory effects under physiological conditions. Our results demonstrated that L-826266 alleviated PGE2-induced DO by increasing bladder capacity and reducing NVCs at a relatively low dose (0.1 mg/kg). Under physiological conditions, L-826266 also significantly prolonged the intercontraction interval and enhanced bladder capacity at a relatively high dose (1.0 mg/kg). The greater responsiveness to low-dose L-826266 in SCI-induced and PGE2-induced DO models may be explained by enhanced activation of the PGE2–EP3 signaling axis under pathological conditions. In SCI rats, bladder PGE2 levels were increased and DO was more pronounced, whereas exogenous PGE2 directly induced bladder overactivity in the PGE2-induced DO model. Therefore, low-dose EP3 receptor blockade was sufficient to increase the intercontraction interval and bladder capacity. By contrast, healthy rats have relatively low basal PGE2 levels and no obvious storage-phase DO, leaving a smaller dynamic range for pharmacological improvement.

We then used the acetic acid-induced OAB model to further define the therapeutic boundary of EP3 receptor blockade. Unlike in the PGE2-induced DO model, L-826266 failed to reverse acetic acid-induced urinary frequency. This model-dependent efficacy is consistent with previous findings by McCafferty et al,^12^ who reported that EP3 receptor deletion abolished PGE2-induced bladder overactivity but did not attenuate acetic acid-induced OAB. The lack of efficacy in the acetic acid-induced OAB model may be explained by its distinct model pathological mechanism. Acetic acid shortens the intercontraction interval by activating acid-sensing ion channels, and the intraperitoneal administration of the acid-sensing ion channel antagonist A-317567 effectively attenuates this effect.^32,33^ Therefore, L-826266 exerted minimal effects on acetic acid-induced OAB. These findings suggest that the EP3 receptor antagonist may be particularly effective for PGE2-related DO, whereas its therapeutic effect for DO induced by other stimuli remains to be further explored.

In addition to functional abnormalities, inflammatory changes and fibrotic remodeling are important pathological features of SCI-associated bladder dysfunction. In this study, we further evaluated bladder histopathology after EP3 antagonist administration. Acute L-826266 treatment did not obviously aggravate urothelial edema or fibrotic remodeling in the bladder, suggesting that short-term EP3 receptor blockade did not cause apparent histopathological deterioration. However, it did not produce a statistically significant histopathological improvement, which is not unexpected because bladder fibrosis is a chronic remodeling process. Whether long-term EP3 receptor blockade can modify SCI-associated inflammation or fibrosis requires further investigation.

This study had several limitations. First, the complete SCI rat model does not fully replicate the complex pathophysiology of human SCI. Second, only the acute effects of L-826266 were evaluated. Long-term studies are needed to determine whether EP3 receptor blockade can modify bladder inflammation, fibrotic remodeling, and sustained downstream signaling pathways in chronic SCI. Third, systemic L-826266 administration cannot distinguish bladder-specific effects from actions on neural components of the micturition pathway. Moreover, EP3 receptor blockade may affect multiple cell types, including urothelial cells, neural cells, smooth muscle cells, and immune cells. Additional approaches, such as localized drug delivery or tissue-specific genetic manipulation, are required to clarify the relative contributions of peripheral vs central EP3 signaling and the precise cellular mechanisms of EP3 receptor antagonism. Finally, DSD is an important contributor to urinary retention after SCI. Because external urethral sphincter activity was not recorded after L-826266 administration, the effect of EP3 receptor blockade on voiding coordination remains unknown. Future studies assessing DSD will further clarify the therapeutic benefit of EP3 receptor blockade for SCI-induced bladder dysfunction.

In conclusion, targeted antagonism of the EP3 receptor effectively suppresses SCI-induced DO. These findings deepen our understanding of prostaglandin signaling in the pathogenesis of DO and support the potential of EP3 receptor antagonist as novel therapeutic agents for DO treatment.

## Data Availability

The data that support the findings of this study are available on request from the corresponding author.
